# Cullin1对肺腺癌A549和H1395细胞生物学特性的影响

**DOI:** 10.3779/j.issn.1009-3419.2021.104.04

**Published:** 2021-02-20

**Authors:** 静怡 刘, 姗娜 苏, 慧洁 何, 慧敏 王, 冬 张

**Affiliations:** 1 014010 包头，内蒙古科技大学包头医学院第一附属医院 The First Affiliated Hospital of Baotou Medical College, Inner Mongolia University of Science and Technology, Baotou 014010, China; 2 014010 包头，内蒙古科技大学包头医学院 Baotou Medical College, Inner Mongolia University of Science and Technology, Baotou 014010, China

**Keywords:** Cullin1, 肺肿瘤, 生物学特性, Cullin1, Lung neoplasms, Biological characteristics

## Abstract

**背景与目的:**

Cullin1是Cullin家族中有代表性的一员，对细胞周期、转录和信号转导相关蛋白泛素化起重要作用，Cullin1与多种恶性肿瘤的发生发展有着密切的联系。本研究旨在探讨Cullin1对肺腺癌A549和H1395细胞生物功能的影响。

**方法:**

实时荧光定量聚合酶链式反应（polymerase chain reaction, PCR）检测肺腺癌细胞（A549、H358、H1395、H1650）及人正常肺上皮细胞BEAS-2B中Cullin1 mRNA表达，采用siRNA技术干扰Cullin1 mRNA相对高表达的肺腺癌细胞；采用四甲基偶氮唑盐比色法（methyl thiazolyl tetrazolium assay, MTT）、流式细胞术及Transwell实验检测细胞增殖、细胞周期分布、细胞早期凋亡及侵袭和迁移能力；采用Western blot检测基质金属蛋白酶-2（matrix metalloproteinase-2, MMP-2）、基质金属蛋白酶-9（matrix metalloproteinase-9, MMP-9）、组织基质金属酶抑制剂-1（tissue inhibitor of metalloproteinase-1, TIMP-1）、细胞周期蛋白D1（Cyclin D1）、细胞周期蛋白E2（Cyclin E2）、p21和p27蛋白的表达水平。

**结果:**

与BEAS-2B细胞相比，肺腺癌细胞中Cullin1 mRNA均呈高表达，尤其在肺腺癌A549和H1395细胞中相对表达量较高（*P* < 0.05）；干扰Cullin1后肺腺癌细胞的增殖能力受到了抑制，G_1_期细胞增多，S期细胞数目减少，肺腺癌细胞早期凋亡率明显升高（*P* < 0.05）；肺腺癌细胞的侵袭及迁移能力下降（*P* < 0.05）；干扰Cullin1后MMP-9、MMP-2、Cyclin D1及Cyclin E2蛋白表达量减少（*P* < 0.05），而TIMP-1、p21和p27蛋白表达量增多（*P* < 0.05）。

**结论:**

干扰Cullin1后可抑制肺腺癌A549和H1395细胞的增殖、侵袭和迁移，Cullin1在肺腺癌中发挥促癌作用。

## Abstract (en)

**Background and objective:**

Cullin1 is a representative member of the Cullin family, and it plays an important role in the ubiquitination of cell cycle, transcription and signal transduction related proteins. Cullin1 is closely related to the occurrence and development of a variety of malignant tumors. The aim of this study is to investigate the effects of Cullin1 on biological function of lung adenocarcinoma A549 and H1395 Cells.

**Methods:**

The expression of Cullin1 mRNA was detected by quantitative Real-time polymerase chain reaction in lung adenocarcinoma cells (A549, H358, H1395, H1650) and human normal lung epithelial cells BEAS-2B, siRNA technology was used to interfere with lung adenocarcinoma cells with relatively high expression of Cullin1 mRNA; cell proliferation, cell cycle distribution, early cell apoptosis, invasion and migration ability were detected by methyl thiazolyl tetrazolium assay (MTT), flow cytometry and Transwell experiment; Western blot was used to detect the expression levels of matrix metalloproteinase-2 (MMP-2), matrix metalloproteinase-9 (MMP-9), tissue inhibitor of metalloproteinase-1 (TIMP-1), Cyclin D1, Cyclin E2, p21 and p27.

**Results:**

Compared with the BEAS-2B cell, Cullin1 mRNA was highly expressed in lung adenocarcinoma cells, especially in lung adenocarcinoma A549 and H1395 cells (*P* < 0.05). The proliferation ability of lung adenocarcinoma cells was inhibited after interference with Cullin1, and the number of cells in G_1_ phase increased, the number of cells in S phase decreased, and the early apoptosis rate of lung adenocarcinoma cells is significantly increased (*P* < 0.05); The invasion and migration ability of lung adenocarcinoma cells decreased (*P* < 0.05). After interference with Cullin1, the protein expression of MMP-9, MMP-2, CyclinD1 and CyclinE2 decreased (*P* < 0.05), while the expression of TIMP-1, p21 and p27 protein increased (*P* < 0.05).

**Conclusion:**

Interference with Cullin1 inhibits the proliferation, invasion and migration of lung adenocarcinoma A549 and H1395 cells, Cullin1 plays a role in promoting cancer in lung adenocarcinoma.

肺癌是全球癌症死亡的主要原因^[1]^，随着工业化、城市化及环境污染肺癌病因也变得更加复杂，由于早期临床表现缺乏特异性，同时缺乏敏感的筛查指标，大部分患者明确诊断时已处于晚期，失去手术治疗的机会，同时放疗、化疗毒副作用较大，不能高度特异性地杀伤肿瘤细胞，且容易复发和远处转移，故患者的预后较差^[2]^。Cullin1蛋白是Cullin家族的一员，目前在人类基因组中，Cullin蛋白家族共有8个成员，Cullin1为SCF复合体（Skp1-Cul-F-box）的重要组成部分，可介导参与细胞周期进程中许多蛋白质的蛋白酶体的降解，一旦Cullin1的调节机制发生异常，SCF复合体功能也相应发生变化，使癌细胞发生积累或肿瘤抑制因子的过度降解，导致细胞的恶性转化和肿瘤发生^[3, 4]^。近年来研究表明Cullin1与恶性肿瘤的生物学行为密切相关，已有研究表明在肾癌、乳腺癌、结肠癌中异常表达^[5-7]^，而肺癌中少有报道，本研究拟探讨Cullin1对肺腺癌A549和H1395细胞生物学特性的影响。

## 1 材料与方法

### 1.1 材料

肺腺癌细胞（A549、H358、H1395、H1650）及人正常肺上皮细胞BEAS-2B（中国科学院干细胞库），RPMI-1640培养基、0.25%胰酶（美国Hyclone公司产品）、胎牛血清（杭州四季青生物工程公司），TRIzol试剂（上海Invitrogen公司），siRNA Cullin1（上海吉玛制药技术有限公司），Lipofectamine 2000转染试剂（赛默飞世尔科技公司），四甲基偶氮唑盐比色法（methyl thiazolyl tetrazolium assay, MTT）试剂（北京中杉金桥生物技术公司），Transwell（上海碧云天公司），基质金属蛋白酶2（matrix metalloproteinase-2, MMP-2）抗体、基质金属蛋白酶9（matrix metalloproteinase-9, MMP-9）抗体、组织基质金属蛋白酶抑制剂-1（tissue inhibitor of metalloproteinases-1, TIMP-1）抗体、Cyclin D1抗体、Cyclin E2抗体、p21抗体、p27抗体（上海贝晶生物技术有限公司），逆转录试剂盒、实时定量聚合酶链式反应（polymerase chain reaction, PCR）试剂盒SYBR Premix Ex Taq、细胞周期检测试剂盒、Annexin V-FITC/PI凋亡检测试剂盒和内参β-actin抗体（北京瀚海拓新生物技术有限公司）。

### 1.2 细胞培养

将肺腺癌细胞（A549、H358、H1395、H1650）及人正常肺上皮细胞BEAS-2B分别置于含有10%胎牛血清的RPMI-1640培养基和LHC-9培养基中，放置于37 ℃、5%CO_2_的细胞培养箱中培养，取对数生长期的细胞进行实验。

### 1.3 细胞株筛选

利用TRIzol分别提取肺腺癌细胞（A549、H358、H1395、H1650）和BEAS-2B细胞中RNA，按照逆转录试剂盒说明书将提取的RNA反转录成cDNA，根据实时定量PCR试剂盒SYBR Premix Ex Taq进行实验，以GAPDH为内参，采用2^-△△Ct^法计算肺腺癌细胞和BEAS-2B中Cullin1 mRNA表达。选取表达量相对较高的两株肺腺癌细胞进行后续的干扰实验。

### 1.4 转染及分组

将选取的两株肺腺癌细胞置于含有10%胎牛血清的RPMI-1640的培养基中，放置于37 ℃、5%CO_2_的细胞培养箱中培养，取对数生长期的细胞，以每孔2×10^5^个细胞接种于6孔板中，放置于37 ℃、5%CO_2_的细胞培养箱中培养过夜，待细胞培养至对数生长期时，采用瞬时转染，根据Lipo2000说明书，将siRNA转染入细胞中，在37 ℃、5%CO_2_培养箱中进行培养48 h，4 h-6 h换液。分为siRNA干扰组（si-Cullin1组）及对照组（未经转染的肺腺癌细胞，NC组）。

### 1.5 MTT法

取对数生长期的细胞，用0.25%胰酶消化，置成细胞悬液，密度为2×10^4^个/mL，按2, 000个/孔接种于96孔板，置于37 ℃、5%CO_2_培养箱中培养，分别于第1、2、3、4、5天取出96孔板，每孔加入MTT溶液20 μL，继续在培养箱中避光孵育4 h，弃去孔内的培养液，每孔加入150 μL DMSO，置摇床上低速震荡10 min，在酶标仪上测定各孔在490 nm处的OD值。

### 1.6 流式细胞术检测细胞周期

取对数生长期的细胞，用PBS清洗2遍，加入胰酶适度消化细胞，离心5 min，用预冷的PBS重悬细胞，加入预冷的70%乙醇，吹打均匀，放入4 ℃冰箱固定过夜，离心，弃去上清液，用预冷的PBS洗2遍，加入100 μL RNase A，于37 ℃孵育30 min，再加入400 μL PI并充分混匀，4 ℃下避光培养30 min，应用细胞流式仪分析细胞周期。

### 1.7 流式细胞术检测细胞凋亡

取对数生长期细胞，用PBS洗涤2次，加入胰酶消化，1, 000 rpm，离心5 min，弃上清液，将细胞重悬于500 μL的Binding Buffer后，再加入5 μL Annexin V-FITC轻轻混匀，加入5 μL PI染色，避光条件下反应15 min，在流式细胞仪下进行检测。

### 1.8 Transwell侵袭实验

取对数生长期的细胞，调整细胞数为3×10^4^个/mL；在上室内均匀的覆盖稀释好的Matrigel基质胶，在24孔板的底部加入500 μL含10%的胎牛血清的培养基，每孔加入200 μL的细胞悬液，继续在37 ℃、5%CO_2_的培养箱中孵育24 h后取出上室，擦净，将上室加入800 μL的甲醇，室温下固定30 min，用台盼蓝进行染色，上室底部朝上晾干，放置在倒置显微镜下计数。

### 1.9 Transwell迁移实验

取对数生长期的细胞，调整细胞数为2×10^4^个/mL，在24孔板的底部加入500 μL含10%的血清的培养基，在上室中加入200 μL的细胞悬液，继续在37 ℃、5%CO_2_的培养箱中孵育24 h，擦净，固定，染色，计数方法同前。

### 1.10 Western blot

胰酶消化、离心、加入蛋白酶抑制剂以及RIPA裂解液，离心取上清液于EP管中，提取总蛋白，用二奎啉甲酸法（BCA法）测定蛋白浓度；取40 μg蛋白以10%SDS-PAGE电压为100 V，电泳90 min，分离后转到PVDF膜上，用5%脱脂奶粉封闭1 h，加入一抗，4 ℃冰箱孵育过夜；第二日，室温下复温30 min，用TBST洗涤3次，5 min/次，加入二抗，室温下孵育2 h，孵育好后，洗涤方法同上，利用Odyssey红外荧光扫描，以β-actin为内参。

### 1.11 统计学方法

采用统计软件SPSS 23.0统计软件进行分析，数据结果以均数±标准差（Mean±SD）表示，组间差异比较采用*t*检验进行分析；检验水准*α*=0.05，*P* < 0.05为差异有统计学意义，统计图使用GraphPad 8.0软件绘制。

## 2 结果

### 2.1 Cullin1在肺腺癌细胞株及人正常肺上皮细胞BEAS-2B中的表达

采用实时定量PCR检测肺腺癌细胞（A549、H358、H1395、H1650）及人正常肺上皮细胞BEAS-2B中Cullin1 mRNA的表达水平，以人正常肺上皮细胞BEAS-2B为参照，Cullin1 mRNA在肺腺癌细胞中均呈高表达，在肺腺癌A549和H1395细胞中的表达相对较高，差异有统计学意义（*P* < 0.05），因此选取这两株肺腺癌细胞进行后续的实验（图 1）。

**1:**
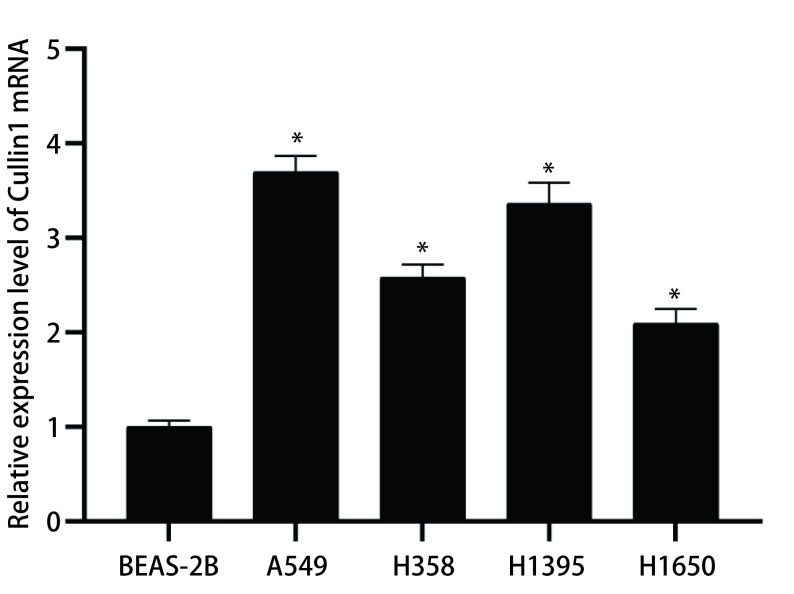
qRT-PCR检测肺腺癌细胞（A549、H358、H1395、H1650）及人正常肺上皮细胞BEAS-2B中Cullin1 mRNA的表达。与人正常肺上皮细胞BEAS-2B相比：^*^P < 0.05。 The expression of Cullin1 mRNA in lung adenocarcinoma cells (A549, H358, H1395, H1650) and human normal lung epithelial cells BEAS-2B by qRT-PCR. Compared with human normal lung epithelial cells BEAS-2B: ^*^P < 0.05. qRT-PCR: quantitative real time polymerase chain reaction.

### 2.2 MTT实验

肺腺癌A549和H1395细胞经过转染后，第4天开始si-Cullin1组A549和H1395细胞OD值低于NC组，差异有统计学意义（*P* < 0.05），第5天si-Cullin1组A549和H1395细胞OD值明显低于NC组，差异有统计学意义（*P* < 0.01）（图 2）。

**2:**
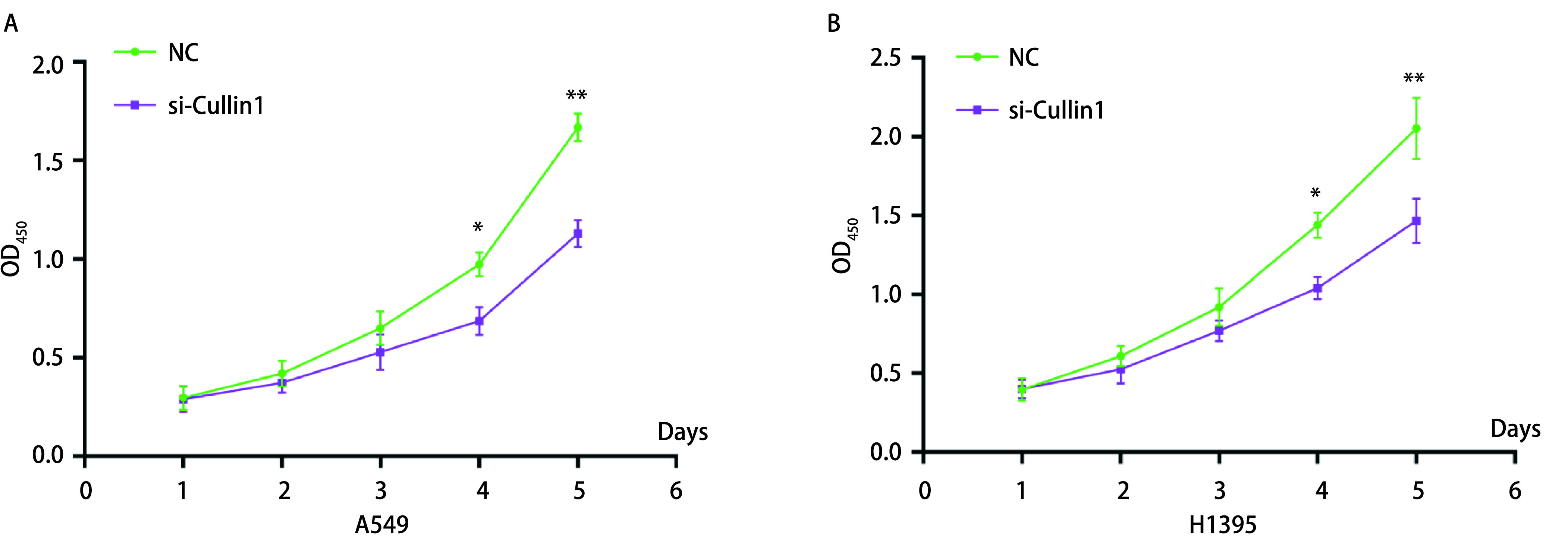
MTT法检测干扰Cullin1后对肺腺癌A549（A）和H1395（B）细胞增殖的影响。^*^P < 0.05，^*^^*^P < 0.01。 The effect of interference with Cullin1 on proliferation of lung adenocarcinoma A549(A) and H1395(B) cells was detected by MTT assay. ^*^P < 0.05, ^*^^*^P < 0.01. OD: optical density; MTT: methyl thiazolyl tetrazolium assay.

### 2.3 干扰Cullin1后对细胞周期的影响

与NC组比较，si-Cullin1组A549和H1395细胞处于G_1_期的细胞增多，S期细胞减少，差异有统计学意义（*P* < 0.05）（图 3A、图 3B）。

**3:**
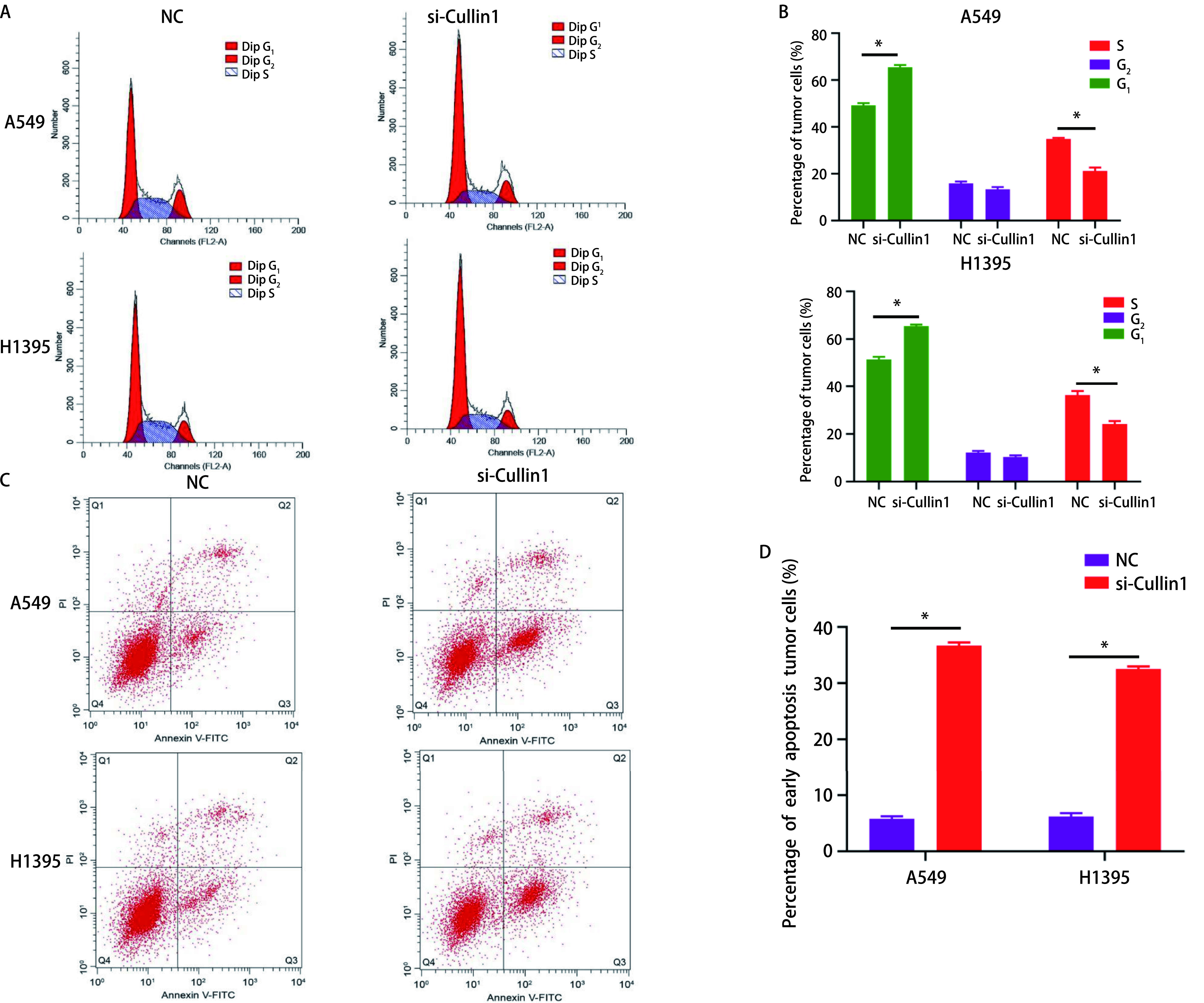
流式细胞术检测干扰Cullin1后对肺腺癌A549和H1395细胞周期及早期凋亡的影响。A：流式细胞术检测细胞周期；B：肺腺癌A549和H1395细胞周期的柱状图比较；C：流式细胞术检测细胞早期凋亡数量；D：肺腺癌A549和H1395细胞早期凋亡率的柱状图比较。^*^P < 0.05。 Flow cytometry to detect the effect of interference with Cullin1 on the cell cycle and early apoptosis of lung adenocarcinoma A549 and H1395. A: Flow cytometry to detect cell cycle; B: Comparison of lung adenocarcinoma A549 and H1395 cells cycle histograms; C: Flow cytometry to detect the number of early apoptosis; D: Comparison of histograms of early apoptosis rate of lung adenocarcinoma A549 and H1395 cells. ^*^P < 0.05.

### 2.4 干扰Cullin1后对细胞早期凋亡的影响

与NC组比较，si-Cullin1组A549和H1395细胞早期凋亡率增加，差异有统计学意义（*P* < 0.05）（图 3C、图 3D）。

### 2.5 Transwell细胞侵袭实验

肺腺癌A549和H1395细胞经Cullin1 siRNA转染后，与NC组相比，穿过Transwell小室的细胞数分别下降57%、42%，差异有统计学意义（*P* < 0.05）（图 4A、图 4B）。

**4:**
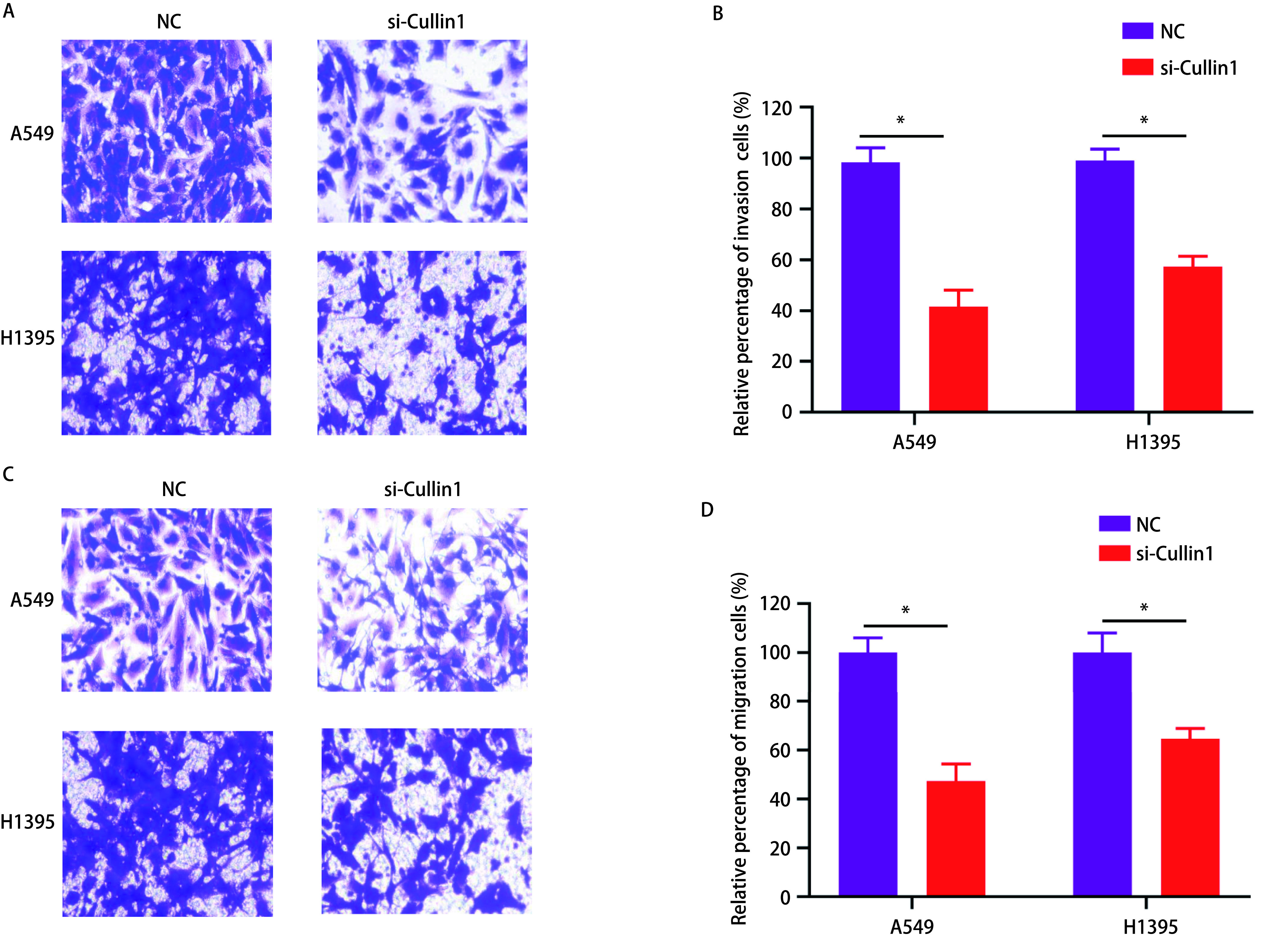
Transwell实验检测干扰Cullin1后肺腺癌A549和H1395细胞侵袭及迁移的能力。A：侵袭实验检测穿过基底膜细胞数量；B：肺腺癌A549和H1395细胞侵袭率的柱状图比较；C：迁移实验检测穿过基底膜细胞数量；D：肺腺癌A549和H1395细胞迁移率的柱状图比较。^*^P < 0.05。 The ability of invasion and migration of lung adenocarcinoma A549 and H1395 cells was detected by Transwell experiment after interference with Cullin1. A: The invasion experiment detected the number of cells passing through the basement membrane; B: Histogram comparison of cell mobility in lung adenocarcinoma A549 and H1395 cells; C: The migration experiment detected the number of cells passing through the basement membrane; D: Histogram comparison of cell mobility in lung adenocarcinoma A549 and H1395 cells. ^*^P < 0.05.

### 2.6 Transwell细胞迁移实验

肺腺癌A549和H1395细胞经Cullin1 siRNA转染后，与NC组相比，穿过Transwell小室的细胞数分别下降52%、35%，差异有统计学意义（*P* < 0.05）（图 4C、图 4D）。

### 2.7 CyclinD1、CyclinE2、p21、p27蛋白表达水平

与NC组相比，肺腺癌A549细胞CyclinD1蛋白表达水平下降55%，CyclinE2蛋白表达水平下降53%，而p21蛋白表达增加30%，p27蛋白表达增加34%，差异有统计学意义（*P* < 0.05）（图 5A、图 5B）。而H1395细胞CyclinD1蛋白的表达水平降低了39%，CyclinE2蛋白的表达水平降低了48%，差异有统计学意义（*P* < 0.05），而p21蛋白表达增加25%，p27蛋白表达增加31%，差异有统计学意义（*P* < 0.05）（图 5C）。

**5:**
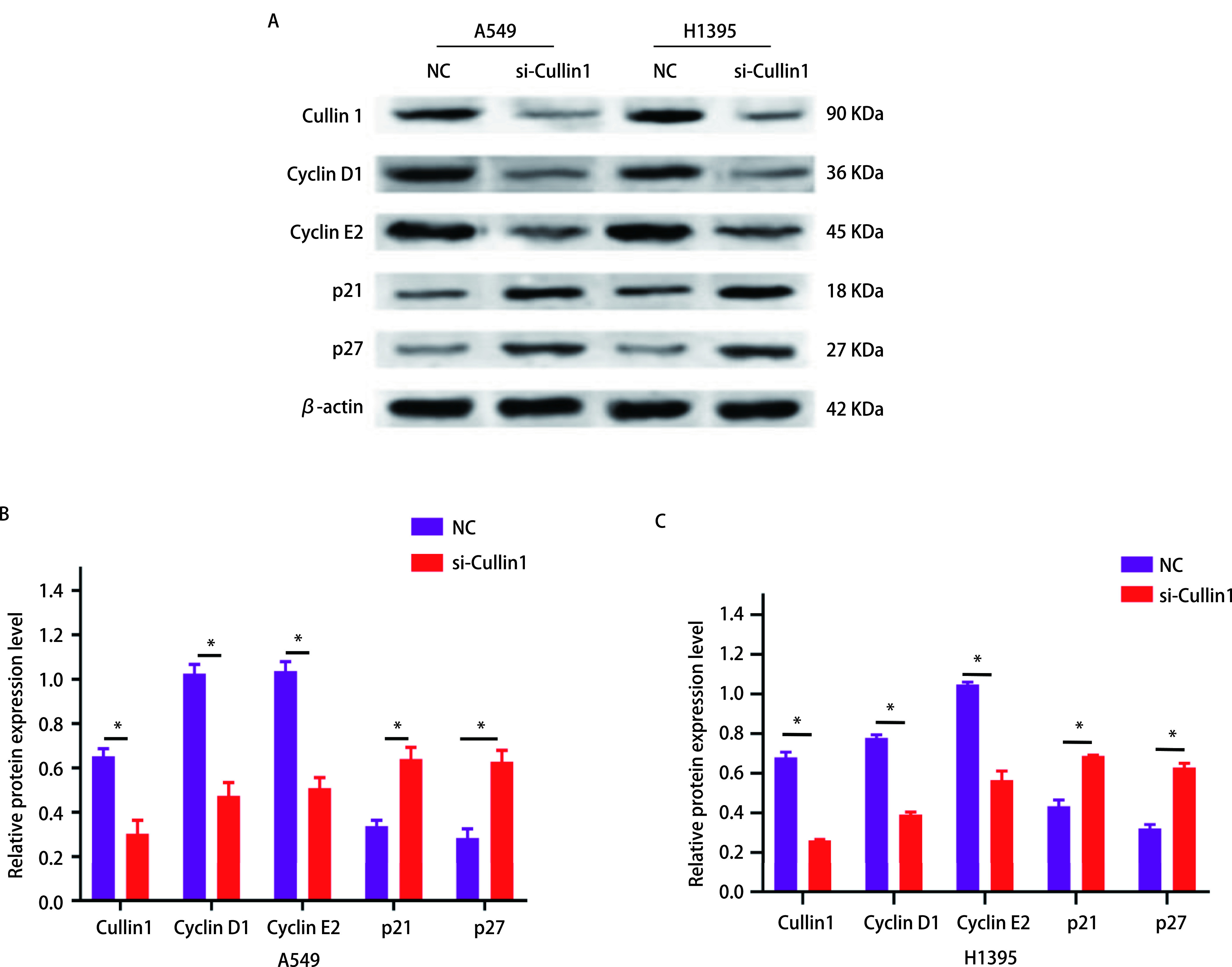
Western blot检测Cyclin D1、Cyclin E2、p21和p27在肺腺癌A549和H1395细胞中的蛋白表达含量。A：Western blot验证CyclinD1、CyclinE2、p21和p27表达；B：肺腺癌A549细胞中相对灰度值表达比较柱状图（^*^P < 0.05）；C：肺腺癌H1395细胞中相对灰度值表达比较柱状图。^*^P < 0.05。 Western blot was used to detect the protein content of Cyclin D1, Cyclin E2, p21 and p27 in lung adenocarcinoma A549 and H1395 cells. A: Western blot to verify the protein expression of Cyclin D1, Cyclin E2, p21 and p27; B: The histogram of relative gray value expression comparison in lung adenocarcinoma A549 cells (^*^P < 0.05); C: The histogram of relative gray value expression comparison in lung adenocarcinoma H1395 cells. ^*^P < 0.05.

### 2.8 干扰Cullin1对MMP-2、MMP-9及TIMP-1蛋白水平的影响

与NC组相比，肺腺癌A549细胞的MMP-9蛋白表达水平降低48%，MMP-2蛋白表达水平降低60%；而TIMP-1蛋白表达增加78%，差异有统计学意义（*P* < 0.05）（图 6A，图 6B）。H1395细胞MMP-9蛋白表达水平降低16%，MMP-2蛋白表达水平降低52%（*P* < 0.05），TIMP-1蛋白表达增加38%，差异有统计学意义（*P* < 0.05）（图 6C）。

**6:**
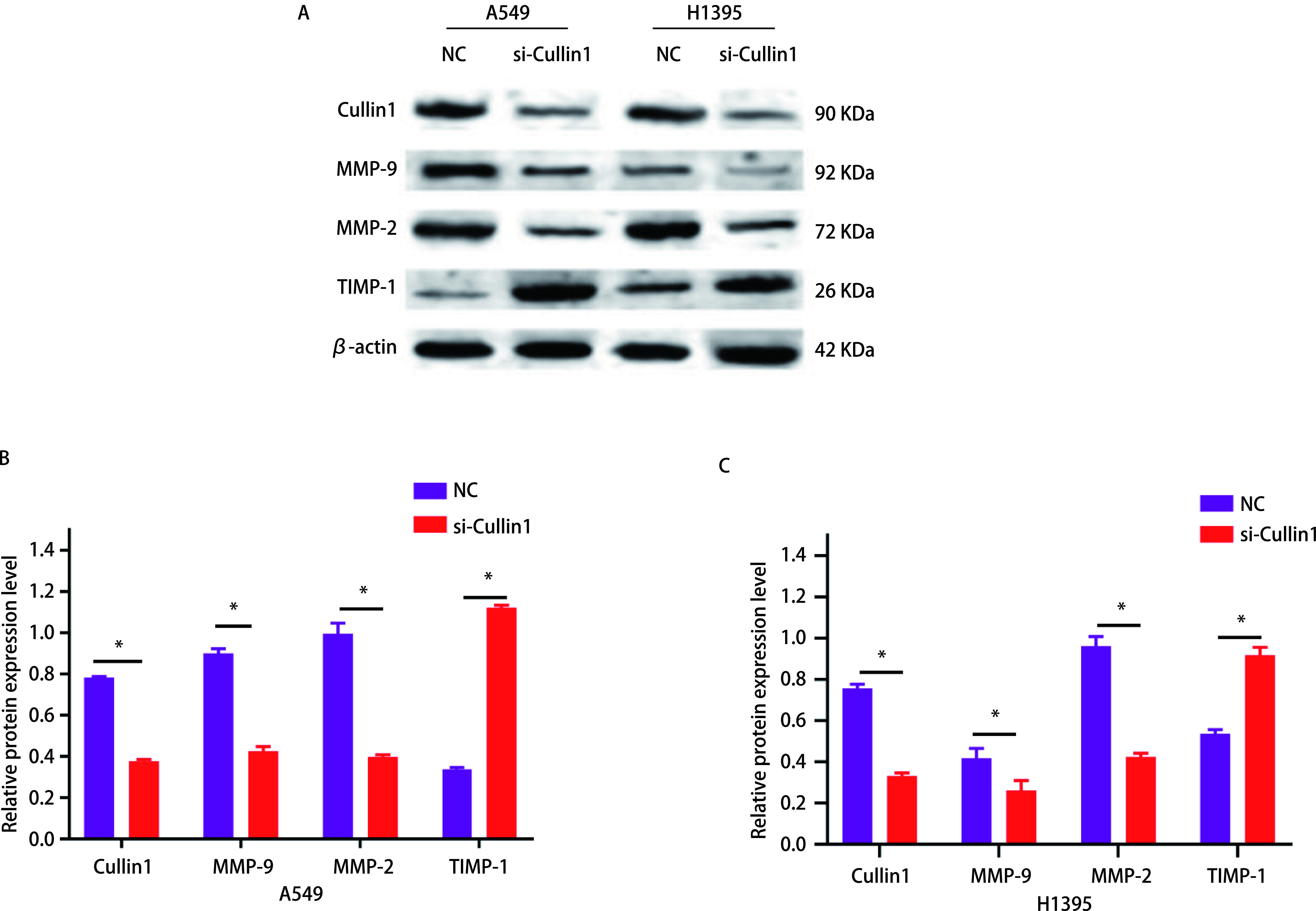
Western blot检测MMP-9、MMP-2和TIMP-1在肺腺癌A549和H1395细胞中的表达蛋白的含量。A：Western blot验证MMP-9、MMP-2和TIMP-1表达；B：肺腺癌A549细胞中相对灰度值表达比较柱状图；C：肺腺癌H1395细胞中相对灰度值表达比较柱状图。^*^P < 0.05。 Western blot was used to detect the protein content of MMP-9, MMP-2 and TIMP-1 in lung adenocarcinoma A549 and H1395 cells. A: Western blot to verify the protein expression of MMP-9, MMP-2 and TIMP-1; B: The histogram of relative gray value expression comparison in lung adenocarcinoma A549 cells; C: The histogram of relative gray value expression comparison in lung adenocarcinoma H1395 cells. ^*^P < 0.05. MMP: matrix metalloproteinase; TIMP: tissue inhibitor of metalloproteinase.

## 3 讨论

近几年，随着肺腺癌相关基因研究不断深入，许多有关肺腺癌生长和发展的基因被发现，更多的治疗方法也不断出现^[8, 9]^。Cullin1蛋白是Cullin家族功能最广泛的成员，是SCF复合体的一种支架蛋白，可以调节多种蛋白因子，如p21、p27和p53等，同时也参与调节细胞周期、信号转导和转录等过程。关于Cullin1与恶性肿瘤之间的研究也越来越多，Min等^[10]^研究发现乳腺癌中Cullin1的表达与预后因素如组织学分级高及p53蛋白表达相关，与治疗标志物如ER阴性、HER2阳性密切相关，Cullin1高表达与总生存率低显著相关，同时Cullin1调节乳腺癌细胞的增殖、迁移和侵袭。Bai等^[11]^发现干扰Cullin1后可以使胃癌细胞增殖能力下降，将细胞周期阻滞在G_1_期，可以降低细胞黏附能力，而癌细胞黏附于靶组织是癌转移侵袭和迁移的关键步骤。Liu等^[12]^发现Cullin1高表达与肝癌的TNM分期及不良预后相关。研究发现Cullin1的高表达可使大肠癌细胞停滞在细胞周期G_1_期，同时能够促进肿瘤的增殖和侵袭，Cullin1高表达与结肠癌淋巴结转移及不良预后有关^[13, 14]^。

肿瘤细胞的主要特征是有较高的增殖能力，且远远超过正常细胞^[15]^。在本研究中，实时定量PCR结果显示肺腺癌细胞（A549、H358、H1395、H1650）中Cullin1 mRNA表达水平较人正常肺上皮细胞BEAS-2B增高，尤其在肺腺癌细胞A549和H1395中最显著，后续实验采用肺腺癌A549和H1395细胞进行。通过MTT法和流式细胞术检测细胞的增殖、细胞周期及早期凋亡情况，结果发现干扰Cullin1表达后，肺腺癌A549和H1395的增殖能力受到明显的抑制，G_1_期细胞数量较对照组明显增加，S期细胞数量减少，细胞早期凋亡率明显增加，干扰Cullin1表达后可使细胞周期进程停滞在G_1_期，从而抑制了细胞的增殖。细胞异常的增殖通常与细胞周期的调节相关，主要与细胞周期蛋白依赖性激酶（cyclin-dependent protein kinases, CDKs）有关，CDKs是丝氨酸/苏氨酸蛋白激酶家族的成员，在控制细胞分裂和调节转录方面发挥着重要作用，其活性主要受细胞周期蛋白调控^[15]^。细胞周期蛋白属于一个传统保守的蛋白家族，在特定的细胞周期阶段表达，从而调节CDKs的活性^[16]^。有文献^[17]^报道细胞周期蛋白D（Cyclin D）、细胞周期蛋白E（Cyclin E）或者细胞周期蛋白A（Cyclin A）和CDKs形成的复合物可促进细胞周期进程。p21和p27是细胞周期蛋白依赖性激酶家族的成员，它们通过阻止细胞从G_1_期进入S期而发挥抑癌作用，p21和p27的功能是抑制Cyclin D1和Cyclin E2蛋白表达水平^[18, 19]^。Cullin1通过泛素化蛋白水解系统调节Cyclin D1、Cyclin E2及CDKs抑制剂p21和p27的表达，该蛋白水解系统失调可能导致增殖失控^[20]^。本研究通过Western blot实验结果发现，Cyclin D1和Cyclin E2蛋白表达量减少，而p21和p27蛋白表达量增多，表明干扰Cullin1后导致泛素-蛋白酶体系统功能障碍，p21和p27蛋白的积累进一步导致Cyclin D1和CyclinE2蛋白表达量减少。Cyclin D1和Cyclin E2蛋白表达量的减少，使肺腺癌细胞从G_1_期向S期转变的进程受到影响，细胞周期停滞，从而抑制了细胞的增殖。

肿瘤的转移是在一系列复杂的细胞侵袭-转移级联上完成的，通过周围环境侵入局部的细胞外基质（extracellular matrix, ECM）和基质细胞层，在转移部位重新启动增殖程序，从而造成肿瘤的发生^[21]^。基质金属蛋白酶（matrix metalloproteinases, MMPs）属于锌依赖性内肽酶家族，主要作用是破坏ECM，同时使肿瘤血管生成增加^[22]^，肿瘤间质中MMPs水平升高与肿瘤细胞浸润或转移呈正相关^[23]^。MMP-2和MMP-9属于MMPs家族成员，主要作用是降解基底膜和ECM的基本成分IV型胶原，从而促进肿瘤细胞的转移，特别是MMP-2被认为在肿瘤侵袭的初始步骤中起着重要作用；MMP-9在肿瘤的侵袭、转移和血管生成中发挥作用，并介导肿瘤微环境的改变^[24, 25]^。TIMPs是MMPs的天然抑制剂，现已有四位家族成员，分别为TIMP-1、TIMP-2、TIMP-3、TIMP-4，其家族成员均能与MMPs形成1:1的共价复合物，抑制MMPs的活性。TIMP-1不仅有抑制MMPs活性的功能，还参与多种生物学活动，包括细胞分化、生长、迁移、侵袭、血管生成和凋亡^[26]^。本研究通过细胞侵袭和迁移实验，发现干扰Cullin1后两种肺腺癌细胞（A549和H1395）的侵袭和迁移能力都出现了明显的下降；为了进一步研究其机制，通过Western blot实验结果发现si-Cullin1组细胞的MMP-9、MMP-2的蛋白表达量较对照组下降，而TIMP-1蛋白表达量则增加，这可能是由于TIMP-1蛋白表达的上调抑制了MMP-9、MMP-2活性有关，从而导致侵袭和迁移能力的下降。

综上所述，本研究发现Cullin1在肺腺癌细胞中高表达，干扰Cullin1能明显阻滞肺腺癌细胞的增殖，使细胞周期停滞于G_1_期，同时使肺腺癌细胞侵袭及迁移的能力降低，提示Cullin1可能作为肺腺癌治疗新的潜在靶点。
